# Gene duplication of SNAPC1 generates transcription factors for snRNAs and sex-specific piRNAs

**DOI:** 10.64898/2026.08.31.747621

**Published:** 2026-09-01

**Authors:** Lars Kristian Benner, Margaret R Starostik, Rebecca J Tay, Ayaka Inoki, Mindy Clark, Suhua Feng, Michael C Schatz, Steven E Jacobsen, John K Kim

## Abstract

Piwi-interacting RNAs (piRNAs) are small non-coding RNAs essential for transposon silencing and germline integrity across metazoans. In many species, piRNA expression is sexually dimorphic, yet the molecular mechanisms underlying this sex specificity remain poorly understood. In *Caenorhabditis elegans,* sexually dimorphic piRNA expression is regulated at the transcriptional level. We previously identified SNPC-1.3, a paralog of the small nuclear RNA (snRNA) activating protein complex (SNAPc/SNPC) subunit SNAPC1, as a male-specific piRNA transcription factor. However, the factors governing female piRNA expression remained elusive. Here, we identify SNPC-1.2, a second SNPC-1 paralog, as a female-specific piRNA transcription factor. SNPC-1.2 interacts with the core piRNA transcriptional machinery, binds female piRNA loci, is required for female piRNA expression, and promotes hermaphrodite fertility. In contrast, a third paralog, SNPC-1.1, retains the ancestral SNAPc function in snRNA transcription and is dispensable for piRNA biogenesis. Together, these findings reveal how gene duplication and functional specialization within the *snpc-1* gene family generate specificity factors that direct the core SNAP complex to distinct genomic targets, providing a molecular mechanism for sexually dimorphic piRNA expression while maintaining canonical snRNA transcription.

## Full Text Availability

The license terms selected by the author(s) for this preprint version do not permit archiving in PMC. The full text is available from the preprint server.

